# Systematic Scatterometer Wind Errors Near Coastal Mountains

**DOI:** 10.1029/2019EA000757

**Published:** 2019-10-18

**Authors:** Thomas Kilpatrick, Shang‐Ping Xie, Hiroki Tokinaga, David Long, Nolan Hutchings

**Affiliations:** ^1^ Scripps Institution of Oceanography University of California San Diego California USA; ^2^ Research Institute for Applied Mechanics Kyushu University Kasuga Japan; ^3^ Department of Electrical and Computer Engineering Brigham Young University Provo Utah USA

**Keywords:** scatterometer, orographic wind, remote sensing, lee vortex

## Abstract

Satellite scatterometers provide the only regular observations of surface wind vectors over vast swaths of the world oceans, including coastal regions, which are of great scientific and societal interest but still present challenges for remote sensing. Here we demonstrate systematic scatterometer wind errors near Hawaii's Big Island: Two counter‐rotating lee vortices, which are clear in the International Comprehensive Ocean‐Atmosphere Data Set ship‐based wind climatology and in aircraft observations, are absent in the Jet Propulsion Laboratory and Remote Sensing Systems scatterometer wind climatologies. We demonstrate similar errors in the representation of transient Catalina Eddy events in the Southern California Bight. These errors likely arise from the nonuniqueness of scatterometer wind observations, that is, an “ambiguity removal” is required during processing to select from multiple wind solutions to the geophysical model function. We discuss strategies to improve the ambiguity selection near coastal mountains, where small‐scale wind reversals are common.

## Key Points

- Orographic wind features near Hawaii's Big Island and southern California are absent in satellite scatterometer wind observations
- These systematic scatterometer wind errors near coastal mountains are due at least partially to ambiguity removal errors
- Prior studies based on scatterometer winds therefore likely underestimate the influence of coastal mountains on coastal ocean circulation

## 1 Introduction

Satellite scatterometers provide daily observations of surface winds and surface wind stress over the global oceans. These are the only regular vector wind observations over vast swaths of the global oceans and are therefore vital for climate study (Chelton & Xie, 2010; Xie, 2004) and forecasting applications (Atlas et al., 2001; Chelton et al., 2006). Scatterometers measure the microwave backscatter from gravity‐capillary waves (Chelton & Freilich, 2005), and the surface wind speed and direction are determined by inverting a geophysical model function (GMF). The GMF inversion is nonunique, that is, the scatterometer wind processing generally results in multiple wind vector solutions at each grid point. An “ambiguity removal” scheme is required to select from the available wind vector solutions (Long & Mendel, 1991; Naderi et al., 1991; Ulaby & Long, 2013).

Here we demonstrate systematic wind errors in the lee of Hawaii's Big Island, where westerly wind associated with two lee vortices has been observed from aircraft (Nickerson & Dias, 1981; Smith & Grubisic, 1993) and ships (Patzert, 1970) but is absent in scatterometer wind climatologies. We show that transient Catalina Eddy events in the Southern California Bight, when coastal winds blow poleward, are also absent in scatterometer winds. The wind errors near Hawaii and southern California indicate a systematic problem with the ambiguity removal in regions of strong orographic forcing, where small‐scale wind features are common. Our results therefore imply that wind stress curl in regions of strong orographic forcing is substantially larger than estimated in previous studies (e.g., Chelton et al., 2004; Xie et al., 2001), with consequences for coastal ocean circulation.

Section 2 describes our data and method; section 3 demonstrates the systematic scatterometer wind errors near Hawaii and southern California; section 4 traces the wind errors to ambiguity selection; section 5 discusses strategies for reducing the errors; and section 6 closes with a summary.

## 2 Data and Method

We compare scatterometer wind climatologies from two different instruments to in situ observations. QuikSCAT data are available for 1999–2009: Here we consider both the Remote Sensing Systems (RSS) version 4 QuikSCAT winds (Ricciardulli et al., 2011), available on a 0.25° grid, and the Jet Propulsion Laboratory (JPL) version 4 QuikSCAT winds (SeaPAC, 2018), which are Level 2 (in the satellite swath) winds available at ∼12.5‐km grid resolution. For the wind fields shown in Figures 3a and 5a, we map the JPL QuikSCAT winds to a 0.25° grid by using a weighted smoother with a 40‐km radius, similar to the loess smoother used by O'Neill et al. (2015). We also utilize the RSS version 2.1 Advanced Scatterometer (ASCAT) winds (Ricciardulli & Wentz, 2016), available on a 0.25° grid from 2007 to present, and the Royal Netherlands Meteorological Institute (KNMI) ASCAT Level 2 coastal winds (ASCAT Wind Product User Manual, 2018), which we interpolate to a 12.5‐km grid.

For the Hawaii region, we compare maps of the scatterometer‐derived wind climatology to a ship‐based wind climatology for 1950–2017. Ship observations from the International Comprehensive Ocean‐Atmosphere Data Set (ICOADS) Release 3 (Freeman et al., 2017) are mapped to a 0.15°×0.15° grid, following the method of Tokinaga et al. (2005). We also consider ICOADS sea level pressure (SLP) fields, which we compare to the Hawaii Regional Climate Model (HRCM; Zhang et al., 2012). The HRCM is a regional atmospheric model simulation of the Hawaii region for 1990–2009; HRCM uses the Weather Research and Forecasting (WRF) model (Skamarock et al., 2008) with the lateral boundaries set to MERRA reanalysis values.

For the Southern California Bight analyses in section 3.2, we interpolate the Level 2 JPL QuikSCAT winds to National Data Buoy Center (NDBC) locations to avoid smoothing out small‐scale wind features. We refer to the NDBC buoys by their last two digits, for example, buoy 25 refers to NDBC buoy 46025. The NDBC buoy winds are available at hourly intervals, as averages over 8‐min periods.

## 3 Systematic Scatterometer Wind Errors

### 3.1 Hawaii's Big Island

Easterly trade winds blow over the subtropical North Pacific and encounter the Hawaiian Islands, producing wakes behind each island (Ma et al., 2009; Xie et al., 2001; Yang et al., 2008). Hawaii's Big Island is the largest island in the chain, ∼150 km across. Its topography is dominated by two mountains, Mauna Kea (4,205 m) and Mauna Loa (4,170 m), which divert the trade wind flow and cause counter‐rotating vortices to form on the lee (west) side of the island (Smith & Grubisic, 1993; Yang et al., 2008). Westerly reverse flow at the surface is associated with the lee vortices, extending 50‐ to 100‐km offshore.

The lee vortices and reverse flow appear in the ship‐based climatology of Patzert (1970), extending to roughly 156.5°W, though it is not clear how many observations Patzert used. The lee vortices and reverse flow also appear in aircraft observations taken at 45‐m height (Nickerson & Dias, 1981) and at 450‐m height (Smith & Grubisic, 1993); the aircraft observations show the lee vortices extending to roughly 157°W.

**Figure 1:**
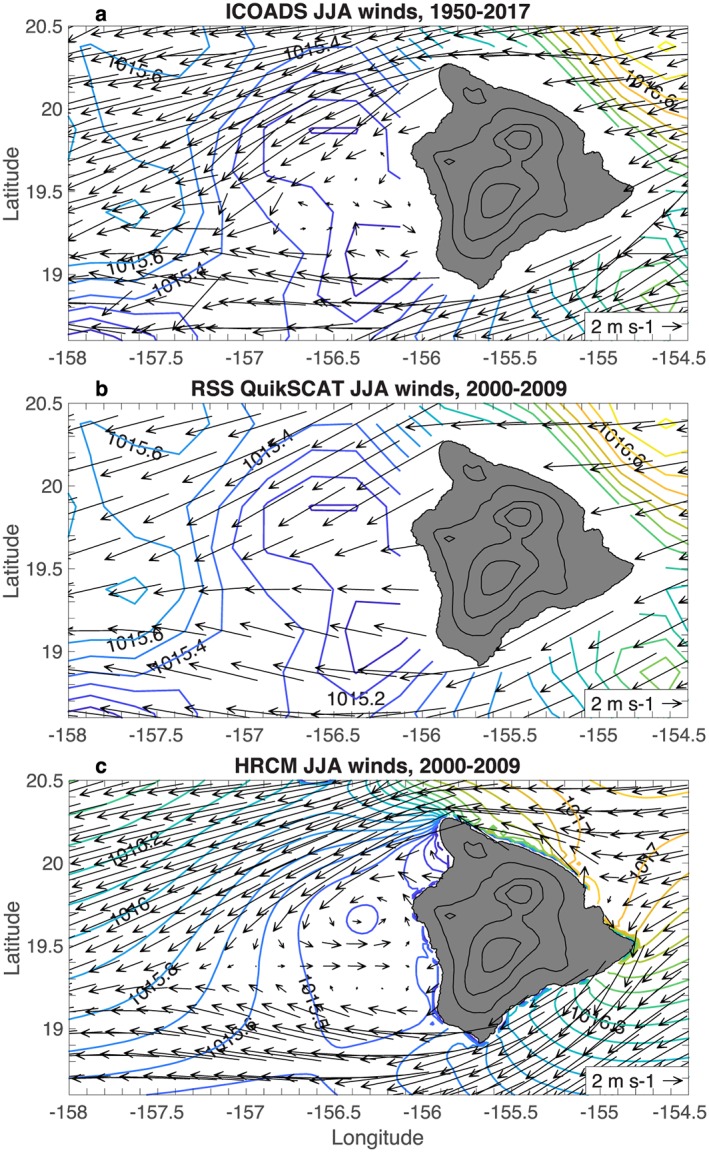
Scatterometer JJA wind climatologies: (a) ICOADS 0.15° winds, 1950–2017; (b) RSS QuikSCAT 0.25° winds, 2000–2009; and (c) HRCM winds subsampled at 15‐km resolution, 2000–2009. Sea level pressure contours from ICOADS (a,b) and HRCM (c) are overlaid (CI=0.1 hPa). Land elevation is contoured in black over Big Island (CI=1 km). Counter‐rotating vortices and reverse flow are visible in the lee of Big Island in ICOADS (a) and HRCM (c) but absent in QuikSCAT (b). HRCM = Hawaii Regional Climate Model; ICOADS = International Comprehensive Ocean‐Atmosphere Data Set; JJA = June–August; RSS = Remote Sensing Systems.

**Figure 2:**
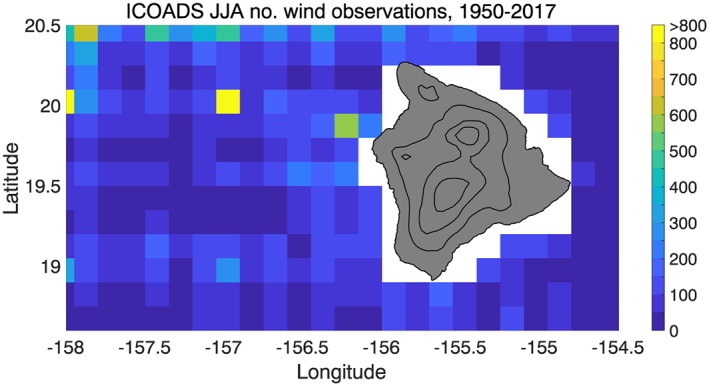
Number of ICOADS wind observations in each 0.15° grid box during the JJA season, 1950–2017. The sampling decreases rapidly with distance from the west side of Big Island. ICOADS = International Comprehensive Ocean‐Atmosphere Data Set; JJA = June–August.

We have compiled ICOADS ship observations of surface winds and pressure on a 0.15° grid for 1950–2017; the lee vortices and reverse flow appear in the June–August (JJA) climatology (Figure 1a). Similar to Patzert (1970), the reverse flow extends to 156.5°W, a narrower zonal extent than that seen in aircraft observations. Some of the spatial variability in the ICOADS climatology is due to the limited sampling; there are a few hundred observations in each 0.15° grid box within ∼60 km of the west coast of Big Island, but the sampling drops to <50 observations in each grid box as one moves farther away (Figure 2). (ICOADS wind studies typically use much coarser grids; e.g., Tokinaga et al., 2005.)

ICOADS SLP contours reveal minima in the lee region with amplitudes of ∼0.2 hPa, consistent with the lee vortices. Note that we average the ICOADS SLP in space with a 9‐point smoother. We do not expect the lee vortices to be fully geostrophically adjusted, as the length scale for an air parcel to reach geostrophic adjustment after passing an obstacle is *L*_*g*_=*U*/*f*≈200 km (e.g., Kilpatrick et al., 2014), where a typical trade wind speed is *U*= 10 m/s and the Coriolis parameter is *f*≈5×10^−5^s^−1^. The length scale *L*_*g*_ is larger than the 50‐ to 100‐km scale of the lee vortices. Indeed, Smith and Grubisic (1993) argue that ageostrophic accelerations are responsible for the high wind speeds between Maui and Big Island.

**Figure 3:**
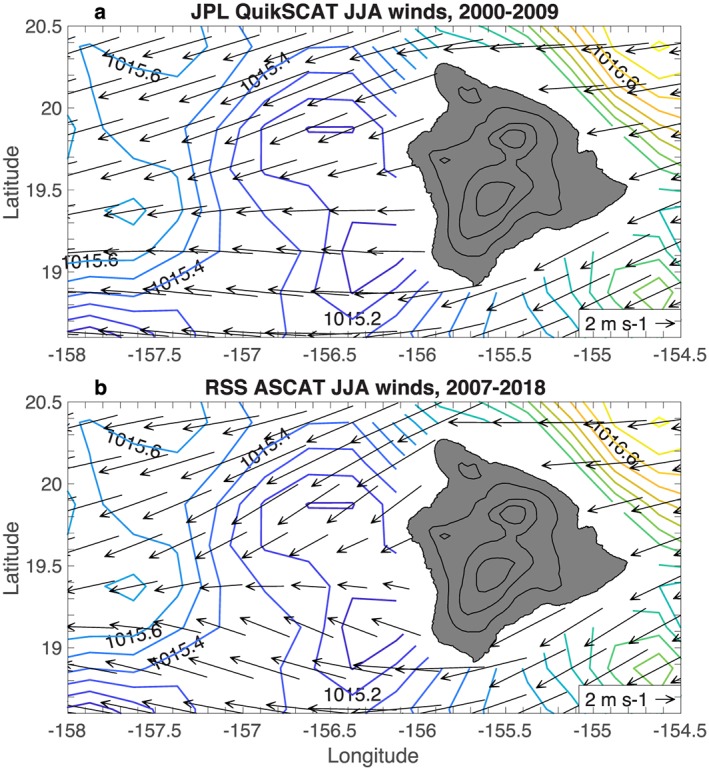
Scatterometer JJA wind climatologies: (a) JPL QuikSCAT version 4.0, 2000–2009; and (b) RSS ASCAT, 2007‐2018. The ICOADS JJA sea level pressure climatology (1950‐2017) is contoured in each panel (CI=0.1 hPa). Land elevation is contoured in black (CI=1 km). The orographically induced reverse flow in the lee of Big Island is absent in both panels. JJA = June–August; JPL = Jet Propulsion Laboratory; RSS = Remote Sensing Systems.

In contrast to the ICOADS winds, the lee vortices and reverse flow are absent in the RSS QuikSCAT JJA wind climatology for 2000–2009 (Figure 1b). The QuikSCAT winds show a deceleration of the easterly trade winds in the lee of Big Island, but no reversal. The reverse flow is also absent in the JPL QuikSCAT version 4 winds (Figure 3a). The absence of the reverse flow in QuikSCAT winds was noted by Hitzl et al. (2014), who hypothesized that this error is due to land contamination of the scatterometer microwave signal. However, the wind errors extend too far from the coast to be blamed on land contamination and are instead due at least partially to ambiguity removal errors (section 4).

**Figure 4:**
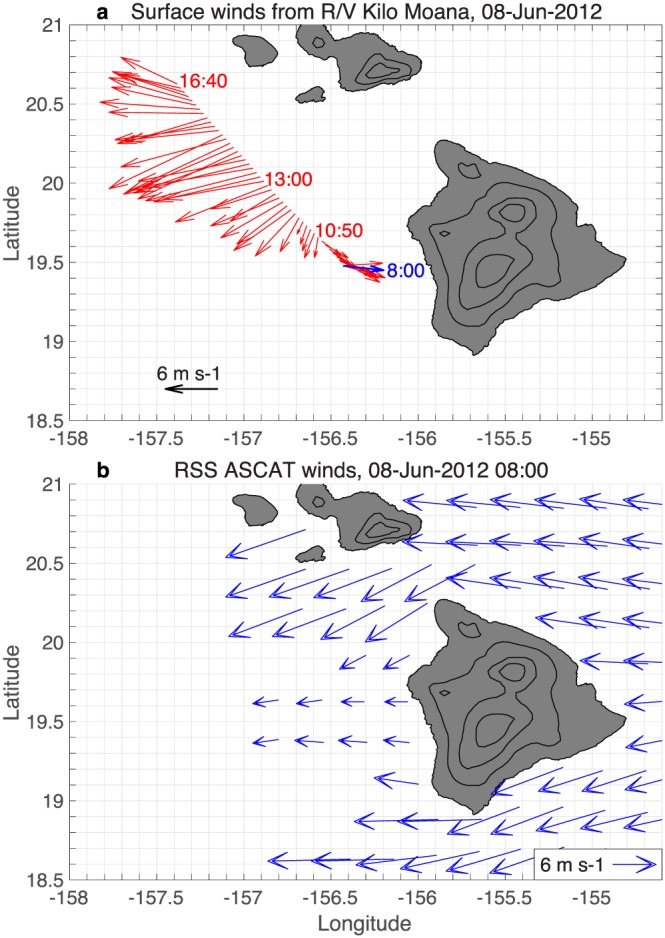
Surface winds on 8 June 2012 from (a) the R/V Kilo Moana and (b) RSS ASCAT. The time (UTC) of the winds in (a) is labeled, with the 8:00 winds shown in blue. The ship wind observations are plotted in 1 hr increments from 6:00 to 9:00 and in 10‐min increments from 9:00 to 16:40. The ASCAT pass (b) is at 8:00; rain‐flagged winds are omitted. Land elevation is contoured in black (CI = 1 km). Reverse flow in the lee of Big Island is clear in (a) but absent in (b). ASCAT = Advanced Scatterometer; RSS = Remote Sensing Systems.

The lee vortices and reverse flow region are also absent in the RSS ASCAT JJA wind climatology for 2007–2018 (Figure 3b), again pointing to a systematic error in the scatterometer wind processing. The 8 June 2012 8:00 UTC ASCAT pass can be compared to shipboard observations from the R/V Kilo Moana, which was transiting from Big Island toward Maui; the ship shows 5 m/s westerly winds at 156.4°W, while ASCAT shows easterly winds (Figure 4). The KNMI ASCAT wind climatology also does not capture the reverse flow region (Figure 10c), despite using a different processing methodology that combines background numerical weather prediction (NWP) winds with scatterometer ambiguities in a variational approach (Stoffelen & Anderson, 1997; Vogelzang et al., 2009).

**Figure 5:**
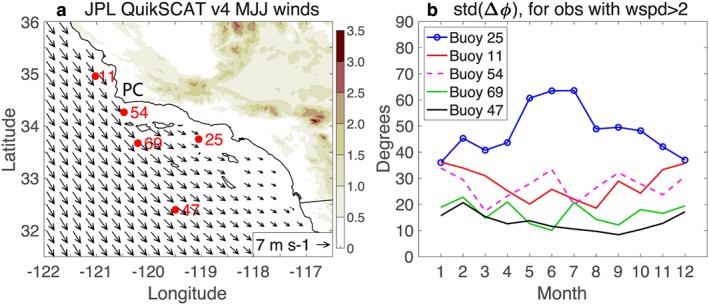
(a) JPL QuikSCAT wind climatology for MJJ, with land elevation (km) in shade. National Data Buoy Center buoy locations are marked in red and Point Conception is labeled “PC.” (b) Standard deviation of the wind direction difference Δ*ϕ* between the buoy wind and the collocated QuikSCAT wind for the buoys marked in (a); observations with wind speeds below 2 m/s are omitted. Large QuikSCAT wind direction errors are found at buoy 25, especially in MJJ, likely due to Catalina Eddy events. JPL = Jet Propulsion Laboratory; MJJ = May–July.

The HRCM model winds show the lee vortices, with reverse flow extending to ∼157°W (Figure 1c). HRCM also shows a local minimum in surface pressure for the northern vortex, with an amplitude of 0.1 hPa. The agreement between the ICOADS ship‐based winds, HRCM model winds, and aircraft observations (Nickerson & Dias, 1981; Smith & Grubisic, 1993) suggests that the lee vortices are real and that the scatterometer winds contain a systematic error.

### 3.2 Southern California Bight

The Southern California Bight is another region with strong orographic wind forcing. The coastline bend at Point Conception and coastal orography shield the inner part of the bight from the upwelling‐favorable winds that prevail in the immediate regions to the north and south (Figure 5a), resulting in strong wind stress curl in the bight (Bakun & Nelson, 1991; Koracin et al., 2004; Winant & Dorman, 1997). This wind stress curl is important for forcing the ocean circulation in this region (Bray et al., 1999; Di Lorenzo, 2003). Figure 5a shows the QuikSCAT surface wind climatology for May through July (MJJ), near the peak of the upwelling season at this latitude (Kilpatrick et al., 2018).

A cyclonic mesoscale atmospheric circulation can form above the bight, known as the Catalina Eddy (Bosart, 1983; Mass & Albright, 1989). During Catalina Eddy events, which can last from a few hours to a few days, coastal winds blow poleward, while offshore winds blow equatorward; an animation of GOES‐16 cloud imagery from 30 May 2018 that shows a Catalina Eddy event is included in the Supporting Information. Catalina Eddy events often result in enhanced cloud cover and cooler air temperature over land (Mass & Albright, 1989; Wakimoto, 1987) and are therefore of interest to weather forecasters.

Here we compare the JPL QuikSCAT version 4 winds to NDBC buoys in the bight. We compute the difference in the wind direction, Δ*ϕ*, between the QuikSCAT surface wind and the buoy wind. To minimize the impact of low‐wind speed observations, we limit ourselves to observations when both the QuikSCAT wind speed and the buoy wind speed exceed 2 m/s. We utilize the QuikSCAT wind at the grid points that contain the buoys shown in Figure 5a and compare each QuikSCAT wind observation to the buoy wind at the nearest available time (within 1 hr).

Figure 5b shows the seasonal cycle of the wind direction difference (Δ*ϕ*) standard deviation, which we refer to as the wind direction error, at the five buoy locations. (The Δ*ϕ* mean, or wind direction bias, is ∼5° or less at all buoys except buoy 54, where the bias ranges from 5° –11° [not shown].) QuikSCAT winds at offshore buoys 69 and 47 have direction errors <20°, within the typical range for scatterometer wind observations (e.g., Ebuchi et al., 2002). However, QuikSCAT winds at buoy 25 in the inner bight have direction errors that exceed 60° during MJJ, before decreasing through the autumn and reaching a minimum below 40° in December–January. QuikSCAT winds at buoy 11, north of Point Conception, and buoy 54, in the Santa Barbara Channel, have direction errors of ∼30° with little seasonal variation.

**Figure 6:**
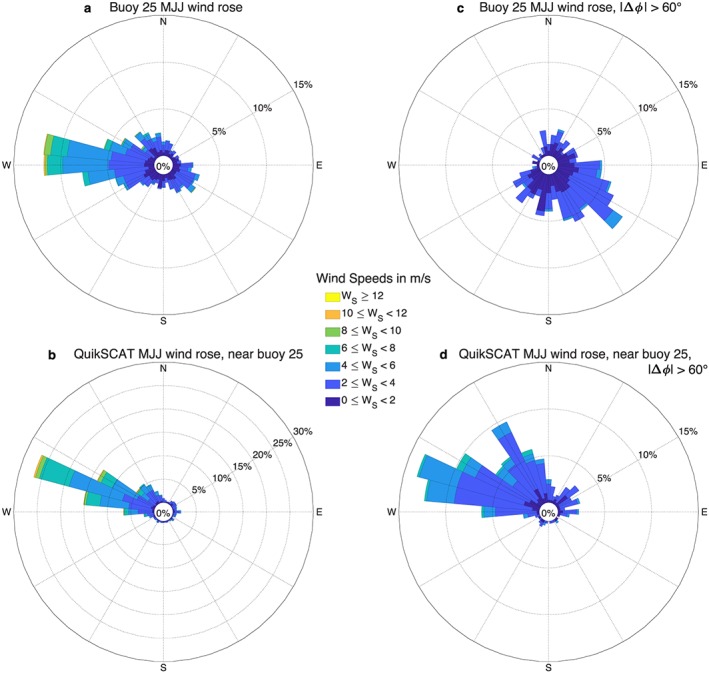
Wind rose, or histogram showing the direction from which winds blow, for buoy 25 MJJ winds (a,c) and collocated JPL QuikSCAT winds (b,d). Wind roses for all MJJ winds with buoy and QuikSCAT collocations available within 1 hr are shown in (a) and (b). Wind roses for the subset of MJJ winds with a wind direction difference |Δ*ϕ*| >60° are shown in (c) and (d). The buoy 25 wind rose (a) has a secondary maximum blowing from the southeast that is absent in the QuikSCAT wind rose (b). For |Δ*ϕ*| >60°, southeasterly buoy winds are dominant (c), while QuikSCAT winds remain westerly, indicating that QuikSCAT winds are not detecting Catalina Eddy events. MJJ = May–July.

What causes the large QuikSCAT wind direction errors in the inner part of the Southern California Bight? Figure 6 shows wind roses, or directional wind histograms, for collocated buoy and QuikSCAT winds at the location of buoy 25. The MJJ buoy winds have a dominant westerly wind direction, with a secondary southeasterly maximum (Figure 6a). In contrast, QuikSCAT shows winds blowing steadily from WNW (Figure 6b), with a secondary maximum notably absent. Wind roses for the subset of collocated observations with |Δ*ϕ*| >60° show buoy winds blowing primarily from the southeast (Figure 6c), while QuikSCAT winds still blow from WNW (Figure 6d), indicating a systematic QuikSCAT wind direction error when buoy winds are southeasterly. The 60° threshold is chosen as the approximate one standard deviation level during MJJ (Figure 5b).

**Figure 7:**
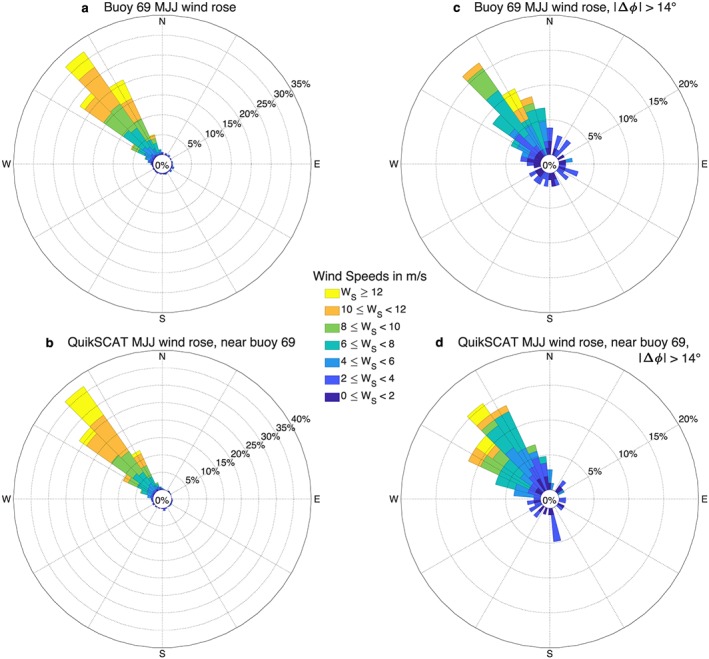
Wind rose for buoy 69 MJJ winds (a,c) and collocated JPL QuikSCAT version 4.0 winds (b,d). Wind roses for all buoy MJJ winds with QuikSCAT collocations available within 1 hr are shown in (a) and (b). Wind roses for the subset of MJJ winds with a wind direction difference |Δ*ϕ*| >14° are shown in (c) and (d). The consistent northwesterly wind direction across samples indicates that there are no systematic QuikSCAT wind direction errors at this location. MJJ = May–July.

MJJ winds at the location of buoy 69, which is 110 km from buoy 25 (cf. Figure 5a), blow steadily from the northwest, whether one plots all observations (Figure 7a,b) or the subset of observations with |Δ*ϕ*| >14° (Figure 7c,d). Note that 14° is approximately one standard deviation of Δ*ϕ* at this location (Figure 5b). The consistency in wind direction across samples indicates that there are no systematic wind direction errors at the location of buoy 69.

**Figure 8:**
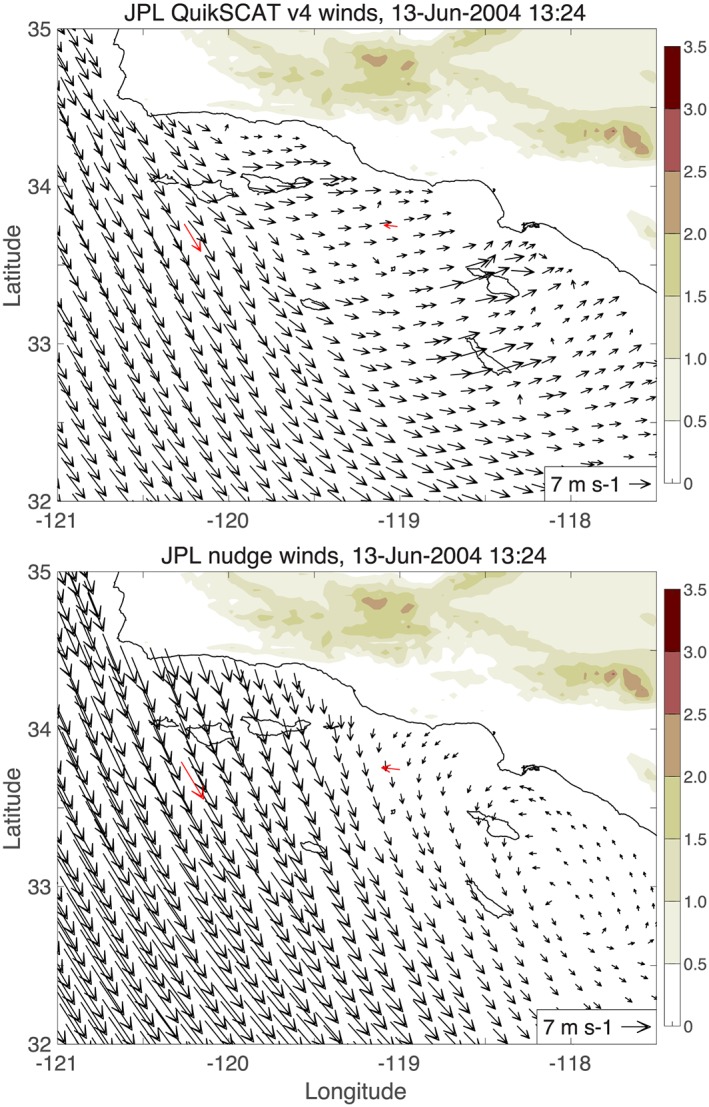
(a) JPL QuikSCAT version 4.0 winds and (b) nudge winds in the Southern California Bight, 13 June 2004 13:24 UTC. The QuikSCAT winds in (a) are Level 2 winds at the full 12.5‐km grid resolution provided by JPL. Buoy 69 (120.2°W) and buoy 25 (119°W) winds at 13:00 UTC are shown in red. The land elevation (km) is shaded. The QuikSCAT winds have a ∼180° direction error near buoy 25. The nudge winds show a Catalina Eddy feature that is absent in the QuikSCAT winds. JPL = Jet Propulsion Laboratory.

Buoy 69 shows steady northwesterly MJJ winds compared to the more variable MJJ winds at buoy 25. Together, Figures 6a and 7a imply that systematic QuikSCAT wind direction errors occur when offshore winds are northwesterly and winds in the inner bight are southeasterly, that is, when there is a mesoscale wind reversal. An example of such a reversal occurred on 13 June 2004: Strong northwesterly winds are seen offshore in QuikSCAT winds (Figure 8a) and at buoy 69, but QuikSCAT does not show the wind reversal over the bight that is observed at buoy 25.

## 4 Ambiguity Selection and Nudge Winds

Scatterometer observations of microwave backscatter are fit to a GMF; inversion of the GMF results in multiple ambiguous solutions, which have similar wind speeds but differ in direction. To select a single wind direction a “nudging field” (usually coarser in resolution, e.g., NWP winds) is used to select an initial wind field from the ambiguous estimates. Nudging is done to ensure general wind trends are present. However, due to the coarse resolution of the nudging field, small‐scale features (e.g., lee vortices and reverse flow) may not be adequately represented, because the nudging field is too coarse to resolve them.

After nudging, the wind field is processed using a median filter‐based ambiguity selection scheme to ensure consistency while preserving wind fronts (Schultz, 1990; Shaffer et al., 1991). At each wind vector cell, the median filter scheme selects the ambiguity that is closest to the circular median direction of the surrounding wind vectors; a 7 × 7 grid point stencil is typically used for this smoothing operation (e.g., Tang et al., 2004). The wind field is then updated, and the process is iterated to convergence. The median filter scheme works well for large‐scale flows but filters out real wind features that are smaller than 25 grid points, even if they are represented in the nudge field. This may be problematic in regions with strong orographic forcing, where fine‐scale wind features are common; in section 5 we discuss the possibility of adapting the median filter stencil size for orographic regions.

**Figure 9:**
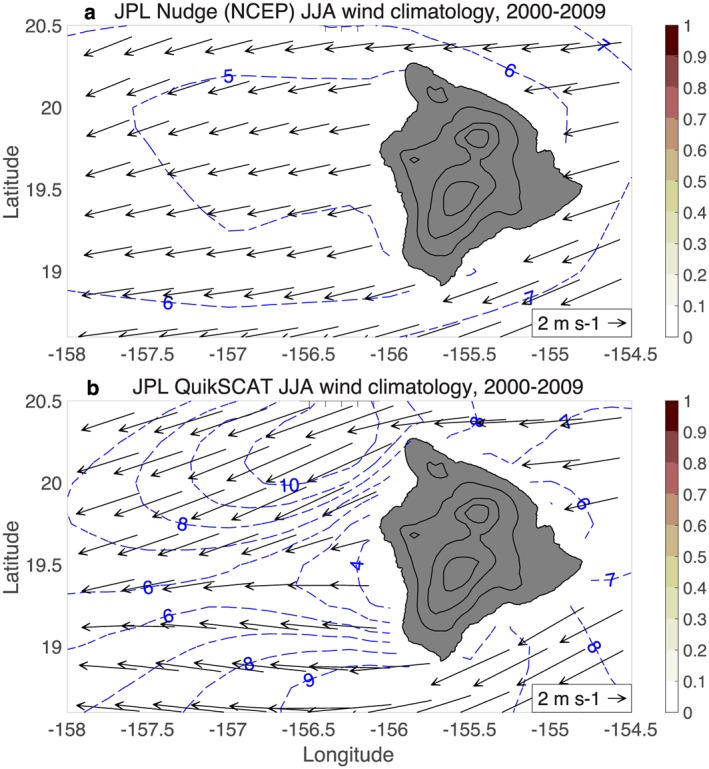
JJA climatology (2000–2009) of (a) nudge wind field used in JPL QuikSCAT processing and (b) JPL QuikSCAT winds. Note that the Big Island wake is completely absent in (a), which is from the coarse NCEP operational analysis. Shading indicates the fraction of the time that westerly (*u* > 0) winds occur, which is roughly 2% in the Big Island wake region for both the nudge winds and JPL QuikSCAT winds. Blue contours show the magnitude of the vector‐averaged wind. JJA = June–July; JPL = Jet Propulsion Laboratory; NCEP = National Centers for Environmental Prediction.

JPL uses relatively coarse (∼1°) National Centers for Environmental Prediction operational analysis winds as the nudge field for QuikSCAT processing (B. Stiles, personal communication, 2019Aug 28). The JJA climatology of the JPL nudge winds is shown for the Big Island region in Figure 9a. We plot the JPL nudge winds on a 0.25° grid, but the actual resolution appears to be much less, with only a slight deceleration of the easterly trade winds visible in the lee of Big Island, and no hint of the lee vortices and reverse flow seen in ship and aircraft observations. Westerly winds in the lee of Big Island occur only ∼2% of the time, which is too low to show any color on the plot. The JPL QuikSCAT winds (Figure 9b) show more of a deceleration in the lee of Big Island, but no lee vortices or reverse flow; the QuikSCAT westerly wind occurrence in the lee region is ∼2%, mirroring the nudge winds. Figure 9 is consistent with JPL ambiguity selection errors in the lee of Big Island being due to nudge winds that do not resolve the orographically induced winds.

**Figure 10:**
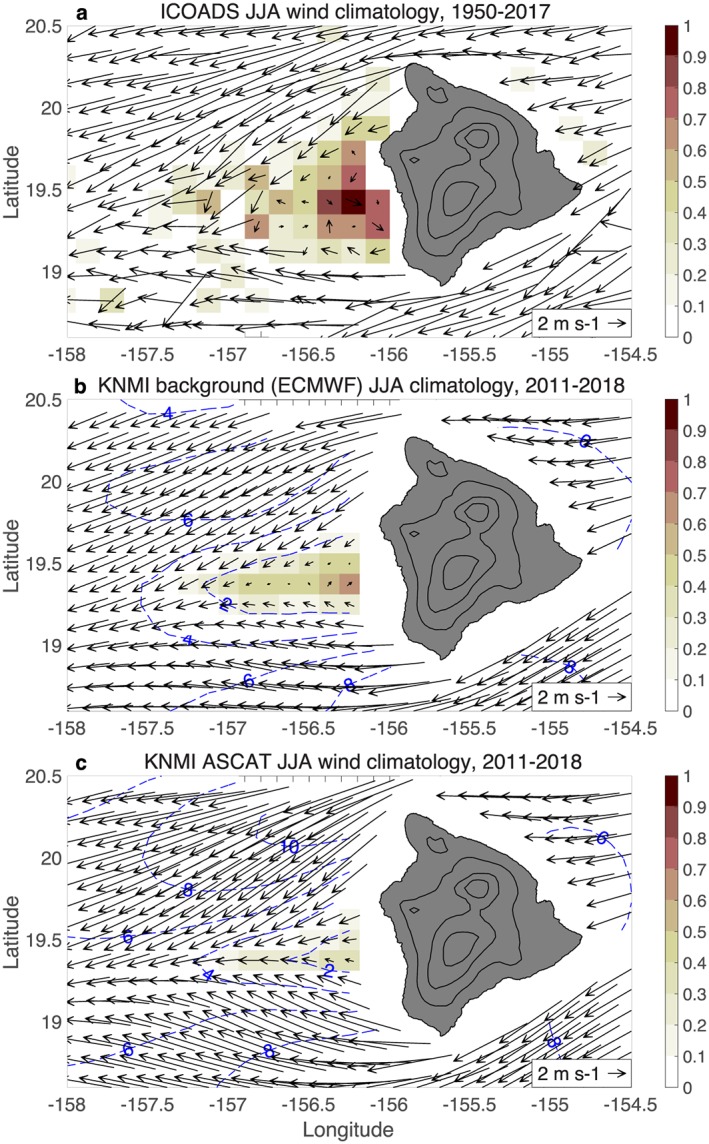
JJA climatology (2000–2009) of (a) ICOADS ship‐based winds; (b) background winds used in KNMI ASCAT processing; and (c) KNMI ASCAT winds. Shading indicates the fraction of the time that westerly (*u* > 0) winds occur, and blue contours show the magnitude of the vector‐averaged wind. The KNMI background winds (b) are high‐resolution ECMWF forecast winds, which have an improved representation of the Big Island wake region compared to the coarse nudge winds in Figure 9a. Notably, the KNMI ASCAT winds (c) have an improved representation of the Big Island wake relative to JPL QuikSCAT winds (Figure 9b). ASCAT = Advanced Scatterometer; ECMWF = European Center for Medium‐Range Weather Prediction; ICOADS = International Comprehensive Ocean‐Atmosphere Data Set; JJA = July–August; KNMI = Royal Netherlands Meteorological Institute.

For comparison, the ICOADS ship‐based JJA wind climatology is plotted again in Figure 10a. The JJA westerly wind occurrence (shaded) exceeds 60% in the lee of Big Island, illustrating that the reverse flow is a fairly persistent feature during summer, as hypothesized by Smith and Grubisic (1993).

The Big Island lee vortices and reverse flow do not appear in JPL's nudge winds or processed QuikSCAT winds. Unfortunately RSS does not provide nudge winds in their QuikSCAT or ASCAT files, so we examine KNMI ASCAT winds instead. KNMI uses a different processing methodology than the JPL/RSS procedure described above, that is, KNMI makes its ambiguity selection according to a variational procedure that incorporates information from a background wind field and from the scatterometer ambiguities (Stoffelen & Anderson, 1997; Vogelzang et al., 2009). Despite the differences from the JPL/RSS processing methodology, in both methodologies, the ambiguity selection is influenced by an external NWP wind field.

KNMI currently obtains background winds from a European Center for Medium‐Range Weather Prediction forecast model with ∼10‐km grid resolution (A. Stoffelen, personal communication, May 2019). The JJA background wind climatology shows light westerly flow in the lee of Big Island, with 40–70% westerly wind occurrence (Figure 10b), which is an improved representation of the Big Island wake compared to the JPL nudge winds. The KNMI ASCAT winds (Figure 10c) also have an improved representation of the Big Island wake compared to JPL QuikSCAT winds, with a stronger deceleration of the easterly trades and 20–40% westerly wind occurrence. Figures 9 and 10 suggest that the KNMI ASCAT winds benefit from the higher‐resolution background winds that better resolve the Big Island wake, though we cannot definitively say that the improvement is due to background winds rather than the variational methodology or other factors.

**Figure 11:**
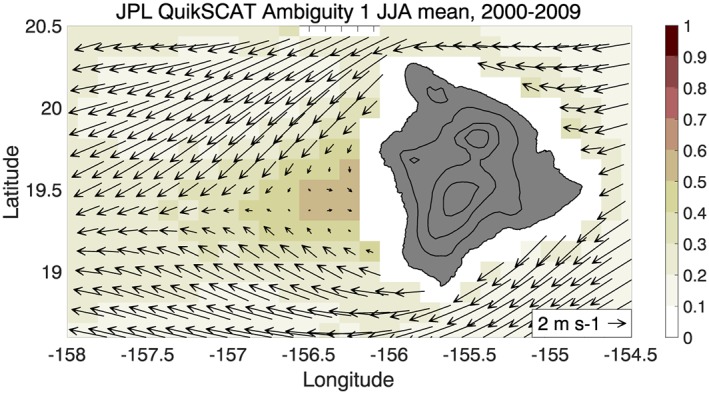
JJA climatology (2000–2009) of JPL QuikSCAT Ambiguity 1, according to the objective function, or fit of microwave backscatter to the geophysical model function. Weak westerly winds are present in the Big Island wake with westerly occurrence of 50–60% of the time. The Ambiguity 1 climatology has a better representation of the Big Island wake than the final processed JPL QuikSCAT winds (Figure 9b), suggesting that the JPL processing often discards the correct ambiguity. JJA = July–August; JPL = Jet Propulsion Laboratory.

Figure 11 shows the climatology of the first ambiguity in the JPL QuikSCAT files, that is, the ambiguity that best fits the GMF according to an objective function. In contrast to the final processed JPL QuikSCAT winds, the Ambiguity 1 climatology shows westerly winds in the lee of Big Island, with 50–60% westerly wind occurrence. Figure 11 implies that QuikSCAT is capable of detecting the reverse flow in the Big Island wake, but the processing methodology is discarding the correct ambiguity.

In the Southern California Bight example shown earlier, the nudge winds (Figure 8b) show a Catalina Eddy, but its structure is inconsistent with the buoy observations (note that the Catalina Eddy is absent in the QuikSCAT winds; Figure 8a). The nudging problem in the Southern California Bight may be one of Catalina Eddy structure and position, whereas the nudge winds do not resolve the Hawaii lee vortices at all.

Lastly, we note that the GMF that relates backscatter to wind is tuned for reporting neutral‐stability, long‐fetch, 10‐m equivalent winds. In the lee of an island, the assumption of neutral‐stability and long‐fetch conditions may be violated; this can result in backscatter/wind modeling errors that contribute to small errors in the reported wind speed that can also affect the reported direction. Previous studies have found larger QuikSCAT errors in coastal regions than in open‐ocean regions (Pickett et al., 2003; Tang et al., 2004), but these studies do not appear to have identified systematic wind errors in coastal regions, which suggests that the GMF is not the primary source of the wind direction errors near coastal mountains that are the focus of our study. However, we cannot rule out the possibility that modifications to the GMF to account for the sea state in coastal regions may be necessary and leave this for future work.

Strategies for improving ambiguity selection near coastal mountains are discussed in section 5.

## 5 Discussion

The systematic scatterometer wind direction errors we have identified near Hawaii and southern California are due at least partially to ambiguity selection (section 4), which consists of nudging and median filter steps. There are several possible strategies for improving ambiguity selection near coastal mountains:

1. *Dynamical downscaling for nudge winds.* For reprocessed scatterometer data sets, one can run a regional atmospheric model for a specific region of interest, obtaining boundaries for the regional domain from global reanalyses. We are actively researching this method for Hawaii's Big Island, utilizing the HRCM WRF model winds for the nudge field. The HRCM WRF model winds resolve the orographically forced mesoscale wind features much better than coarse NWP winds (Figure 1), so we can expect improvement in the climatology of QuikSCAT winds produced using the HRCM WRF winds for the nudge field.
  A drawback of this approach is that one must run the regional atmospheric model for all times that one is interested in obtaining scatterometer winds. Another issue is that the behavior of the orographically forced winds may not be fully determined by the large‐scale conditions, for example, the Big Island wake is somewhat variable (Smith & Grubisic, 1993), so the orographically forced winds in the regional model may not always provide an adequate representation of the orographically forced winds in nature.
2. *Statistical downscaling for nudge winds.* Another approach for producing nudge winds in regions of strong orographic forcing is based on the statistical relationships between winds in the orographic region and winds in the surrounding region. One can determine the statistical relationships from a regional atmospheric model, such as the HRCM winds for Hawaii, and then create a nudge wind field in the orographic region from the NWP model or scatterometer winds in the surrounding region. The advantage of this approach is that one only has to determine the statistical relationships once, and then can apply it ad infinitum. We are actively researching simple regression methods and more sophisticated neural network methods for statistical downscaling.
3. *High‐resolution NWP nudge winds.* JPL uses relatively coarse (∼1°) NWP winds as the nudge field for QuikSCAT processing (Figure 9a). The KNMI ASCAT processing methodology is different from the JPL QuikSCAT processing, but KNMI still uses a background wind field that influences their ambiguity selection. KNMI uses European Center for Medium‐Range Weather Prediction forecast winds with ∼10‐km grid resolution for their background wind field (A. Stoffelen, personal communication); Figure 10 shows that the KNMI background winds and KNMI ASCAT winds have improved representations of the Big Island wake compared to the JPL nudge winds and JPL QuikSCAT winds, respectively. Therefore some improvement in the JPL QuikSCAT winds might be expected by obtaining nudge winds from higher‐resolution NWP models, which have more realistic flows around coastal mountains.
  However, as illustrated by Figure 8, just because an NWP model resolves orographic wind features does not guarantee that those features will be accurate, that is, a high‐resolution NWP model could cause ambiguity selection errors if its orographic wind features are misplaced or distorted relative to the true winds. Another caveat for real‐time applications is that NWP models assimilate scatterometer wind observations, so utilizing those same NWP models for ambiguity selection could lead to scatterometer and NWP wind errors that reinforce each other.
4. *Adjust median filter for coastal regions.* The JPL QuikSCAT processing uses a median filter with a 7 × 7 grid point stencil to spatially smooth the wind field. Since the Level 2 winds have a grid resolution of approximately 12.5 km, the median filter may act too strongly on orographically induced wind features with a 40‐ to 80‐km spatial scale. Therefore it may be beneficial to adapt the median filter stencil size in regions that are known to have a strong orographic forcing. Catalina Eddies were apparently detected in an earlier, specially produced QuikSCAT data set (Hu & Liu, 2002) that was not released to the public; Hu and Liu (2002) do not describe their processing methodology in detail, but they may have used a smaller stencil for the median filter (B. Stiles, personal communication).
5. *Utilize time information in* *σ*_0_ *.*In modern scatterometer processing methodologies, satellite observations of microwave backscatter (*σ*_0_) are fit to the GMF, which is inverted to produce a wind field at each time. For relatively steady mesoscale wind features like in the lee of Hawaii's Big Island, it may be possible to utilize *σ*_0_ observations over a longer period to produce, say, 7‐day or 14‐day averaged winds, where the additional *σ*_0_ observations could be incorporated into the GMF inversion to improve the wind field at lower frequencies. This may require the development of a new GMF inversion technique.

One implication of the wind errors highlighted in this study is that the orographic influence on surface winds is likely substantially larger than in previous scatterometer‐based estimates (e.g., Chelton et al., 2004). Ocean circulation around islands (Basterretxea et al., 2002; Chavanne et al., 2002; Sakamoto et al., 2004; Xie et al., 2001) and capes (Di Lorenzo, 2003; Kilpatrick et al., 2018) is very sensitive to the orographically induced wind stress curl, so the impact of orographic winds on ocean circulation will have to be revisited after corrected scatterometer winds become available. Coupled models (e.g., Seo et al., 2007) may be necessary to tease apart air‐sea feedbacks in these regions.

## 6 Summary

We identify systematic scatterometer wind errors in the lee of Hawaii's Big Island and in the Southern California Bight, two regions with strong orographic influence on surface winds. In the lee of Big Island, QuikSCAT and ASCAT wind climatologies do not capture two lee vortices that appear in ICOADS ship‐based observations and in aircraft observations. In the Southern California Bight, the most recent JPL QuikSCAT (version 4.0) winds do not capture the southeasterly winds associated with transient Catalina Eddy events. The wind errors can be traced to the ambiguity selection that is required due to the nonuniqueness of scatterometer wind observations. Similar scatterometer wind errors are likely near coastal mountains around the world, underscoring the challenge of remote sensing in the coastal zone.

## Supporting information



Supporting Information S1
