# Supplementary material for: Systematic Scatterometer Wind Errors Near Coastal Mountains
Source: Earth Space Sci. 2019 Oct 18;6(10):1900–14. doi: 10.1029/2019EA000757 (PMC6919418; doi:10.1029/2019EA000757)
Supplement: Supplementary file 1 — Supporting Information S1 [file ESS2-6-1900-s001.pdf]

Supporting Information for

Systematic scatterometer wind errors near coastal mountains

Thomas Kilpatrick[1], Shang-Ping Xie[1], Hiroki Tokinaga[2], David Long[3], and Nolan Hutchings[3]

[1]Scripps Institution of Oceanography, University of California, San Diego.
[2]Research Institute for Applied Mechanics, Kyushu University, Kasuga, Japan
[3]Brigham Young University, Provo, Utah.

Additional Supporting Information (file uploaded separately)
Caption for Movie S1 (cf. animation S1.gif)

Introduction
The video in the supporting information (S1.gif) shows GOES-16 cloud imagery, produced by the National Weather Service's San Diego office.

Movie S1
GOES-16 cloud imagery from 30 May 2018 reveals cyclonic motion over the Southern California Bight, indicating a Catalina Eddy event. The GOES imagery is from the 0.64 μm (visible) band, used for daytime monitoring of clouds, and runs from 15:47 UTC to 16:42 UTC.
